# Melt Electrospinning Designs for Nanofiber Fabrication for Different Applications

**DOI:** 10.3390/ijms20102455

**Published:** 2019-05-17

**Authors:** Yasseen S. Ibrahim, Essraa A. Hussein, Moustafa M. Zagho, Ghada G. Abdo, Ahmed A. Elzatahry

**Affiliations:** 1 Materials Science and Technology Program, College of Arts and Sciences, Qatar University, Doha 2713, Qatar; yasseen_ibrahim@hotmail.com (Y.S.I.); eh1604846@student.qu.edu.qa (E.A.H.); 2 School of Polymer Science and Engineering, University of Southern Mississippi, Hattiesburg, MS 39406, USA; moustafa.zagho@usm.edu; 3 College of Pharmacy, Qatar University, P.O. Box, Doha 2713, Qatar; ga1805049@student.qu.edu.qa

**Keywords:** nanofibers, setup, melt electrospinning, laser melt, coaxial, melt electrospinning multitemperature control, gas assist melt electrospinning

## Abstract

Nanofibers have been attracting growing attention owing to their outstanding physicochemical and structural properties as well as diverse and intriguing applications. Electrospinning has been known as a simple, flexible, and multipurpose technique for the fabrication of submicro scale fibers. Throughout the last two decades, numerous investigations have focused on the employment of electrospinning techniques to improve the characteristics of fabricated fibers. This review highlights the state of the art of melt electrospinning and clarifies the major categories based on multitemperature control, gas assist, laser melt, coaxial, and needleless designs. In addition, we represent the effect of melt electrospinning process parameters on the properties of produced fibers. Finally, this review summarizes the challenges and obstacles connected to the melt electrospinning technique.

## 1. Introduction

Since the breakthrough in nanoscience and nanotechnology, many efforts have been devoted to developing simple, reproducible, and variable techniques for the fabrication of nanomaterials. Owing to their unique properties, a huge variety of nanostructured materials and composites have been fabricated for diverse applications such as energy storage, catalysis, food industries, and biomedical applications [1]. One of these nanoscale materials is the one-dimensional (1D) nanomaterial, which has gained a great deal of attention due to its high surface area-to-volume ratio as well as superb performance in many areas [2,3,4]. One-dimensional nanostructures, such as wires, fibers, rods, spirals, and tubes, could be fabricated using different synthetic methods and techniques and varying specific parameters [5,6].

**Figure 1:**
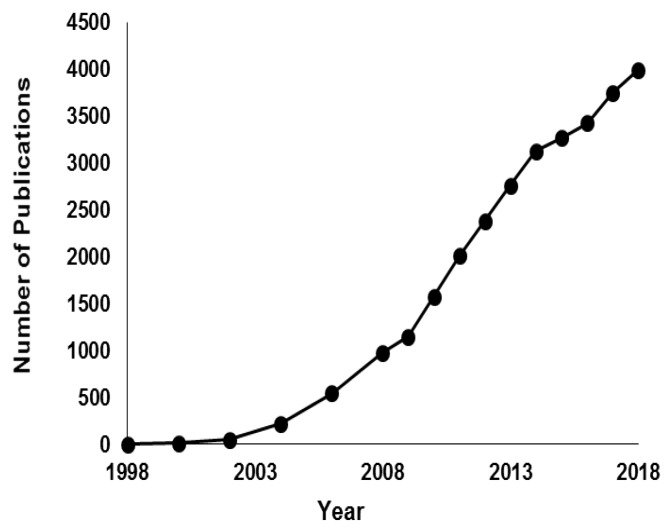
The annual number of English-written journal articles published in the period from 1998–2018, as derived from SciFinder Scholar using the keyword “electrospinning”. As of 6 May 2019, there are 1855 publications.

Nanofibers are considered an important class of 1D nanomaterials and there has been increased interest in their applications in different domains including biomedical/health applications [7,8,9]. Electrospinning has been established and described as the most widely used technique to fabricate nanofibers with uniform shape [10,11,12,13,14]. Furthermore, electrospinning is characterized by simplicity, flexibility, and versatility, which means that it is able to fabricate various nanofibers using a wide range of materials such as polymers, semiconductors, ceramics, and composites [15,16]. Therefore, it is not surprising to find a huge increase in the published journal articles related to electrospinning throughout the last two decades (see Figure 1) due to the reproducibility of the technique as well as ease of production [17].

In spite of all the reported advantages of electrospinning, it still has some limitations concerning the rate and scale of production. To illustrate, for some applications, large scale or massive quantities of the fabricated electrospun nanofibers are required to be produced by the traditional setup, which is relatively time consuming [18]. The literature revealed many studies aimed at mitigating this drawback, either by using double-layered electrospinning setup with multiple nozzles [19,20,21,22] or by using a hollow tube with adjustable lengths and holes to obtain multiple jets, which in turn could speed up the production process [23,24]. In addition, several modifications have targeted the electrospinning process to improve the quality, size, and functionalization of the fabricated fibers. 

The major objectives of this review are: (a) to demonstrate the main types of melt electrospinning technique and briefly discuss the history of each one; (b) to address the development of melt electrospinning design with relevant examples of the produced nanofibers; (c) to shed light on the most recent published applications of electrospun nanofibers using different melt electrospinning processes; and (d) to discuss in brief the challenges of dragging melt electrospinning in practical applications. 

## 2. Melt Electrospinning

The first work on melt electrospinning was done by Charles Norton in a 1936 patent [25,26,27], where he introduced his invention for the fabrication of viscous materials after liquefying them using fusion or adding a volatile solvent. The patent noted that the influence of electrostatic work in conjunction with air blast could lead to the production of fine fibers. Norton’s invention was illustrated in two design forms which are fundamentally alike with minor variations in the materials used such as gums, pitches, and fused glass. In the first design, the container was made of metal or clay which can be heated by an electric heating unit and placed at one to six feet from the target. An electrostatic machine (100 kV) was connected to both the target and the container with positive and negative charges, respectively. The liquid was discharged from the container through a spout, then blown towards the stationary plane target by compressed air. The blown liquid flowed between two parallel plate electrodes (2.2 kV) while being connected to an alternating current of the same charge as the container. The second design, on the other hand, was heated by directing the burning glass flames at the bottom of the container, a rotating cylindrical target. As well, a doctor removed collected fibers and compressed air was subjected perpendicular to the liquid flow path from a rotatory valve instead of the plate electrodes.

**Figure 2:**
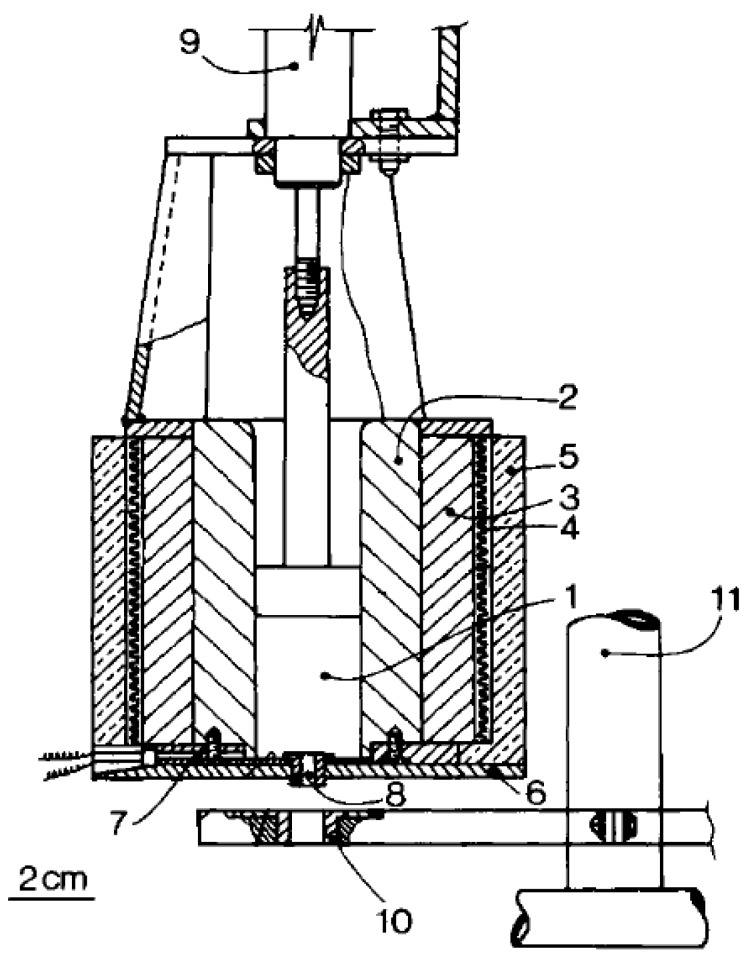
First experimental melt electrospinning setup (**1**) chamber, (**2**) stainless steel wall, (**3**) aluminum jacket, (**4**) heater, (**5**,**6**) insulator, (**7**) thermocouple, (**8**) nozzle, (**9**) air cylinder, (**10**) collector, and (**11**) shaft [28].

In 1981, Larrondo and Manley [28] published the first melt electrospinning experiment as an alternative to solution electrospinning using high-density polyethylene (melt flow index (MFI) = 2), isotactic polypropylene (MFI = 0.5), and polyethylene in paraffin. As illustrated in Figure 2, the melted polymer was placed in a vertical chamber with stainless steel walls. An electric heater was deployed around an aluminum jacket that surrounds the chamber walls, where a thermocouple was used to control the temperature in the chamber. An insulating material covered the sides and the bottom of the device and an air piston cylinder was used to pump out the melt polymer through a stainless steel capillary. The hollow metallic collector palate was placed on a non-conductive lever rod. To control the vertical distance between the capillary and the plate, a shaft connected to the lever rod was fixed. Experiments were performed to determine the minimum voltage at which a stable electrospun jet was formed for each one. During the operations, the temperature of the spun polyethylene in paraffin solutions was about 100 °C, while polyethylene and polypropylenes melts were spun at ranges of 200–220 °C and 220–240 °C, respectively. Additionally, the capillary diameter used was 1 mm for the solutions and 2.2 mm with a 4.5 length/diameter (L/d) ratio of the capillary for the melt. The electrospun fibers showed different surface structures at 6 kV/cm, a shish-kebab-like structure formed from 1% polyethylene in paraffin solution, while the melt polymers formed a random lamellar arrangement and a spherulitic shape for polyethylene and polypropylene, respectively. All the fibers were electrospun at the ambient air temperature without controlling the spun region temperature [28]. 

### 2.1. Multitemperature Control

**Figure 3:**
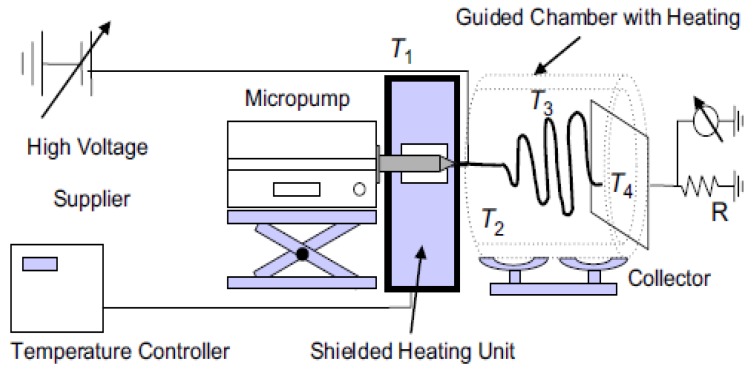
Multi-heating zone melt electrospinning [29].

Zhou et al. [29] presented multitemperature control electrospinning technique as a convenient method to spin melt polymers on a submicron scale fiber as an alternative to solution electrospinning using polylactic acid (PLA). Throughout the proposed design as shown in Figure 3, the melt polymer was subjected to four heating zones: syringe, nozzle, spinning area, and the collector to enhance and control the fiber size and morphology. Several investigations have been reported to study the change on the fiber diameter by altering the nozzle temperature and diameter, spinning temperature, electric field, and flow rate. By taking these parameters into consideration, the PLA melt was successfully electrospun to form 800 nm average fiber diameter at 0.01 mL/min, 20 kV, 0.16 mm nozzle diameter, where the temperature of the syringe, nozzle, spun region, and the collector was 200, 255, 80 and 25 °C, respectively. It is worth noting that the group investigated for the first time the whipping motion of the melt jet, reporting that the melt jet experienced a further thinning just like the solution jet as confirmed on several studies and models [30,31,32,33]. Whereby, varying the applied voltage at 25 °C spinning temperature, the jet motion started to bend more near the collector. On the other hand, at 80 °C a vigorous melt jet motion accorded which increased the residence time of the jet in the spinning region. Proving the merit of the multi-control electrospinning design, numerous studies [34,35,36,37,38] and models [39,40,41] used the same or similar setup designs.

### 2.2. Gas Assist

Zhmayev et al. [42] introduced the concept of gas-assisted melt electrospinning (GAME) as a simple alternative for Zhou’s complicated design, eliminating the difficulty of controlling both the needle and the spinneret temperature to avoid polymer degradation. GAME’s configuration holds a coaxial setup, where the melt flow of polylactic acid (PLA) was spun at 1.67 × 10^−10^ m^3^/s from an inner nozzle with 4.13 × 10^−4^ and 7.18 × 10^−4^ m inner and outer diameters, respectively. The jet of hot air, on the other hand, was blown at 300 m/s from the outer nozzle of 1.194 × 10^−3^ m inner diameter. The temperature of both nozzles was at 483 k and 0.09 m away from the collector which held 2 × 10^4^ V of potential. In terms of process, the initial attenuation force for GAME was based on the electric field, not the air velocity or its temperature, unlike the electro-blowing [43,44,45]. Through the experimental investigation, it was observed that turbulent air applied a drag force resulting in an increase of the production and a decrease of the melt jet diameter by 10% and an extra 20-fold further thinning occurred upon increasing the air jet temperature, such that the melt PLA fiber diameter in 300 k initial air stagnant decreased from 3.5 to 0.18 µm.

**Figure 4:**
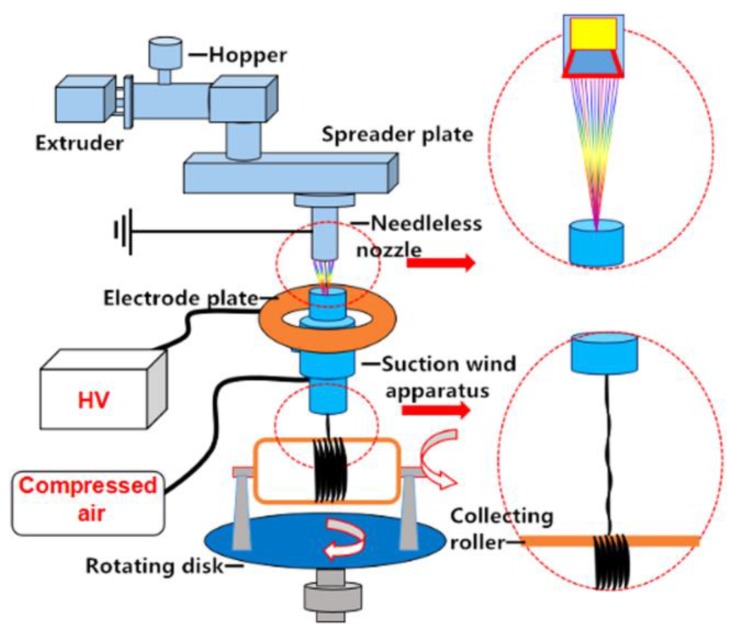
Yarn melt electrospinning: The small dotted circles located around the exit of needles nozzle and the suction wind apparatus are magnified in big dotted circles on the right side, each is pointed out by a red solid arrow to show the role of the suction wind apparatus in combining multiple fibers into one fiber. The hollow red arrows show a vertical and horizontal rotation direction of the collecting roller and rotating disk, respectively [48].

In recent years, the suction wind velocity has been considered a critical parameter on the fiber diameter in yarn manufacturing [46,47]. Xiaolu Ma et al. [48] were able to fabricate yarn fibers with 440 nm average diameter while thinning and twisting multiple jets of polypropylene melt polymer. The proposed design, as represented in Figure 4, was based on a needleless nozzle to increase the production, which held an advantage over the needle creating multiple Taylor cones, a 200 mm diameter collector placed 10 cm away from the needleless nozzle, and a rotating disc that affected the fiber rotating angle. Additionally, a self-designed suction wind device that consisted of an air inlet, annular airside, and air drain allowed the fibers to be drawn from the nozzle and then combined. From the experimental results, the wind speed that showed the higher influence on the fiber diameter as compared with the collector speed under the same applied voltage where the smallest fiber diameter was obtained, was at 30 m/s wind speed. It was also concluded that the twisting angle of the fiber could be controlled by adjusting the speed ratio of the collector to the rotating disk. Pre-investigation, the smallest angle of twisting was 5° at a ratio of 30 and the highest was 43° at a ratio of 2. Even though, several recent methods were developed to manufacture nanofiber yarn, they were only applicable for solution electrospinning [49,50,51,52].

### 2.3. Laser Melt Electrospinning

#### 2.3.1. Spot Laser Melt Electrospinning

PLA was the first polymer to be electrospun using the laser melt electrospinning technique by Ogata et al. in 2007 [53]. Such a method of heating was easily utilized to spin high melting point polymers with minimum thermal degradation and dispersion. Moreover, the laser melt electrospinning did not cause electric discharge as compared with the work published by Lyons et al. [54] and Warner et al. [55]. In Ogata’s experimental work, two-rod samples of 0.5 mm diameter were prepared from 22.3 and 76.9 (g/min) MFI of PLA. The rod samples were then deployed in the laser melt electrospinning at 2–4 mm/s flow. A copper wire was used as a medium to connect the end of the rod sample with an aluminum electrode and high potential difference between the rod and the 1 m/s rotating target was applied. Then, the laser beam was subjected on the other end of the sample rod from three angles by using four mirrors and an absorber. The diameter and wavelength of the laser beam were 5 mm and 10.6 µm, respectively. Additionally, nitrogen gas was used in conjunction with the laser beam to prevent the sample rod from burning. The PLA nanofibers were successfully electrospun from the laser melt electrospinning at a 20 mm collector distance, as the diameter of the low MFI PLA had 804 nm at a laser power and voltage of 17 W and 30 kV. By comparison, the high MFI PLA had a smaller diameter (712 nm) at 13 W and 26 kV, and the MFI of the polymer was considered as a factor affecting the fiber diameter.

The same group continued working on the spot laser melt electrospinning process to spin different polymers: rod polymer—poly(ethylene-co-vinyl alcohol), poly(ethylene terephthalate), and polyalirate by Ogata et al. [56,57]; coated rod polymer—poly(l-lactide) with poly(ethylene-*co*-vinyl alcohol) by Tian et al. [58]; as well as fiber bundle—poly(lactide)/poly(ethylene-*co*-vinyl alcohol) by Shimada et al. [59]. Following the footsteps of this group, further studies have been reported by another group led by Xiuyan Li [60,61,62,63,64] using the same spot laser design.

**Figure 5:**
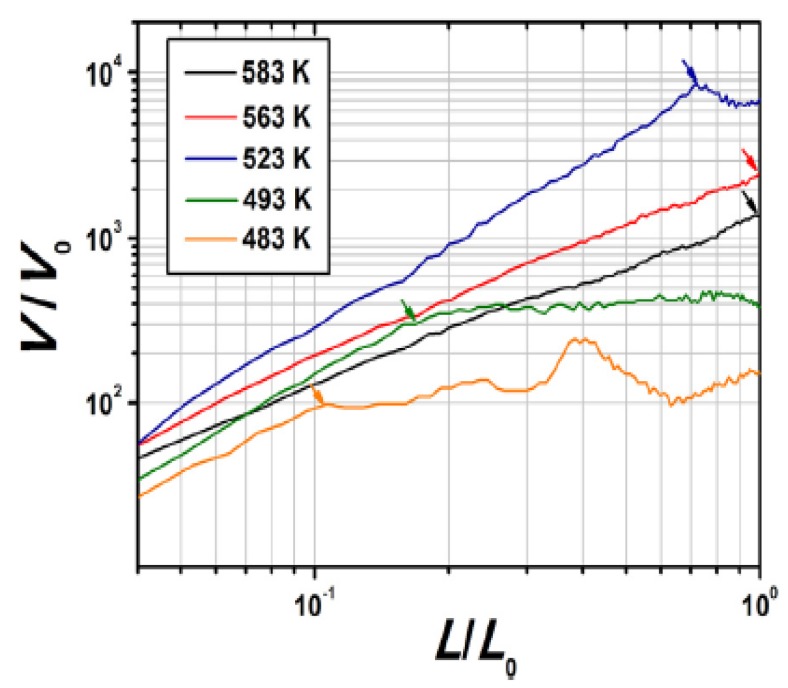
Experimental jet velocity versus spin line for several melt temperatures [26].

Several studies have been published to investigate the process parameters, such as the process temperature, as well as the electro-and thermodynamics of the melt electrospinning. A methodical study by a Xu et al. [26] was published to build a predictive understanding of the basic mechanism that sets the jet motion of the fibers. The authors tackled some addressed problems like molten Taylor cone and thinning in addition to deformation and coiling of the jet. To do so, poly (l-lactic acid) (PLLA) was used in CO_2_ laser-based melt electrospinning, which consisted of a nozzle, a beam splitter, reflector, beam baffle, feeder, high voltage power supply, and laser transmitter, respectively as shown in Figure 5. The feed flow rate and the collector distance were 259 mLh^−1^ and 50 mm, respectively. The fibers were collected on flat target (90 × 90 mm) and a (2 rpm) rotating target with 90 mm outer diameter. From Figure 5, Xu and his co-authors revealed that as the melt temperature increases, the solidification points of the fiber get closer to the collector. It is important to mention, however, as the solidification points decrease, the elongation rate is almost constant, hence, the jet velocity can be calculated by the following equation:

(1) $$v(x)\;=\;\xi \;*\;xv\left(x\right)\;=\;\dot{\varepsilon }*x$$

The authors were able to formulate Equation (3) to estimate the molten fiber diameter before its solidification by combining Equation (1) with Equation (2):

(2) $$v\left(x\right)\;=\;4*Q/\pi *\;d^{2}\left(x\right)v\left(x\right)\;=\;\dot{\varepsilon }*x$$

(3) $$\;d\left(x\right)\;=\;\dot{\varepsilon }/\surd \;\pi \;*\;(Q/\xi {)}^{0.5}\;*\;x^{-0.5}v\left(x\right)\;=\;\dot{\varepsilon }*x$$

where v(x) and d(x) is the velocity and diameter at distance × respectively, ξ is the rate of elongation, and Q is the flow rate.

**Figure 6:**
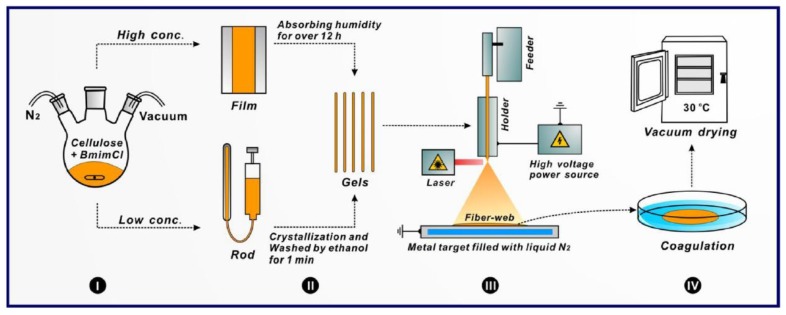
Preparation steps for cellulose fibers: (I) Dissolution, cellulose and BmimCl were mixed in the presence of nitrogen for 2 h to form a homogeneous solution; ( II) gels rods preparation, high and low concentration of cellulose were prepared using film casting and crystallization, respectively to be fed by a holder; (III) melt-laser electrospinning setup, fabricating viscous polymer and collecting freeze fiber at −40 °C; (IV) fibers coagulation, fibers were washed in ethanol bath then dried under vacuum [65].

Most recently, Xu [65] has utilized the laser melt electrospinning process to spin cellulose/BmimCl solution homogeneously mixed with either water or ethanol. A crystallization process and film casting were used to prepare the flexible gel rods of cellulose as represented in Figure 6, unlike melt extrusion [66] and compression molding [56]. The laser-based system consisted of the feeder at 93 µm/s, a laser with a 10 W/cm^2^ output power, and a power supply of 18 kV. The collector (90 × 90 mm) was filled with liquid nitrogen and placed at 5 cm from the holder, where the surface of the collector was about −40 °C. Fine fibers of 1 µm were generated from a high degree of polymerization cellulose when blending with BmimCl for two hours at 110 °C.

#### 2.3.2. Line Laser Melt Electrospinning

A new laser melt electrospinning process was introduced in 2010 by Shimada et al. [67]. It was equipped by a line laser beam to increase the fiber production. The idea of this design was based on projecting a line laser beam on the end of the polymer sheet to produce fibers from a bulk instead of a rod. In the previous spot laser melt electrospinning technique, the rod samples were homogeneously melted from three angles in the following manner: (1) the source laser beam was projected and absorbed partially by the rod; (2) then, the unabsorbed beam was reflected twice to fall back on the sample; and (3) then again, the unabsorbed beam continued on in this manner until it fell on the laser absorber. In the line laser system, however, the line beam was generated by introducing the spot laser beam to an optical system which consisted of three mirrors, a laser homogenizer, a collimator lens, and cylindrical convex and concave lenses. The line laser system was designed to form a top hat shape with uniform intensity over 150 and 2 mm of length and width, respectively. A sheet of poly(ethylene-*co*-vinyl alcohol) (EVOH) and nylon 6/12 were electrospun at a voltage of 40 kV, laser power of 45 W, and collector distance of 100 mm. The study investigated the effect of the flow rate and the thickness of the polymer sheet on the fiber diameter. It was concluded that the average fiber diameter decreases upon decreasing the flow rate or the sheet thickness. It is worth mentioning, in the previous study [59], that an opposite result was obtained concerning the effect of the flow rate on fiber diameter on the rod polymer.

Unlike Lyons et al. [54] and Shen et al. [68], Fujii et al. [69] succeeded to produce nanofibers from polypropylene (PP) with an average diameter using a line-like carbon dioxide (CO_2_) laser beam. Fujii and his team were able to achieve the aim of their study by fabricating (PP/EVOH/PP) three-layer sheets, taking advantage of the melt flow rate between the two polymers. Hence, hollow shape PP fibers were obtained by treating the spun fibers with 2-propanol/water solution to remove the EVOH. A melt press at 5 MPa and 190 °C was performed for 10 min to convert the pellets to sheets (40 mm × 100 mm). The sheets were then placed between the charged copper holders and fed at 4.0 mm/min in the melt electrospinning. To linearly melt the sheet polymers, a beam expander and homogenizer along with two cylindrical lenses were used to switch the spot beam to a line. The fibers were spun from a 100 mm distance and collected on a copper anode target (150 mm × 150 mm × 5 mm). A pure monolayer of each polymer, as well as two-layer (PP/EVOH) and three-layer (PP/EVOH/PP) with three different melt flow rates of pellet PP, were spun with co-spinning material of EVOH in order to demonstrate the change in the fiber diameter. It was noticeable that the thickness diameter of the polymer fibers decreased in the two- and three-layer sheets as compared with the monolayers. Fujii explained that the reason behind the change in fibers diameter was due to the towing effect between the two polymers. 

### 2.4. Coaxial Electrospinning

The first encapsulation melt electrospinning system was invented by McCann et al. [70] to fabricate phase change materials with a coaxial spinneret in the nanoscale. A conventional coaxial electrospinning setup was used, the phase change polymer (octadecane) was held in a glass syringe, where its temperature was set at 68 °C using a heating tape and a thermocouple to maintain its molten state. The glass syringe was attached to a polymer-coated based silica capillary which was embedded in a plastic syringe filled with the polymer solution (PVP/Ti(OiPr)4). Both melt and solution polymers were pumped separately by two pumps at 0.2 mL/h and 0.7 mL/h for the 7% octadecane, and 0.3 mL/h and 0.7 mL/h for the 45% octadecane of the fibers by weight. A high voltage was applied at the metallic needle to spin the polymers on an aluminum foil. 

**Figure 7:**
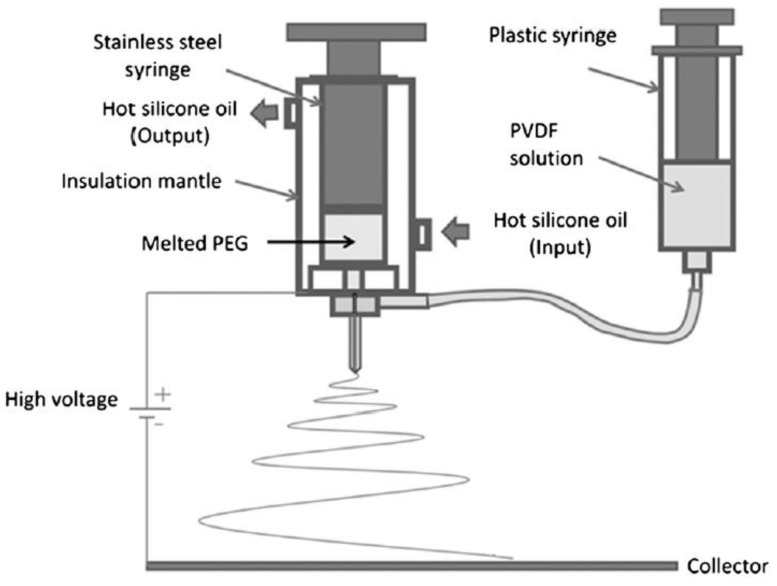
Schematic diagram of melt coaxial electrospinning [71].

Do et al. [71] used polyethylene glycol (PEG)/polyvinylidene fluoride (PVDF) in the melt-solution coaxial system to enhance the results obtained from their previous study [72] using solution coaxial electrospinning. As shown in Figure 7, stainless steel and plastic syringes were filled with melt PEG and 20% of PVDF/*N*,*N*-dimethylformamide (DMF) solution, respectively. The steel syringe maintained a constant temperature (70 °C) through the circulation of hot silicon oil. Both polymers were spun coaxially at 12 kV and 17 cm voltage and collector distance, respectively through an inner diameter of 0.35 and 1.05 mm. While maintaining the flow of the solution polymer (shell) constant at 1.5 mL/h through all the trials, increasing both the molecular weight and feed rate of the core polymer resulted in an increase of the fiber diameter. 

A comparative study can be accomplished between the results of the two studies based on the obtained capacity of the core/shell nanofibers in energy storage. From the Do et al. [71] study, the highest enthalpy ratio was 42.5% for PEG4000 corresponding to 68 J/g latent heat among all the core/shell fibers. On the other hand, McCann et al. [70] obtained value was 114 J/g from 45% octadecane by weight of fibers. The possible explanation is based on the difference in enthalpy between octadecane and PEG. Nevertheless, the obtained results from the melt/solution coaxial electrospinning showed better results than that of others [72,73,74,75]. In the same manner, Li et al. [76] used the same system to spin Crystal violet lactone–bisphenol A–1-tetradecanol mixture/poly (methyl methacrylate) (CBT/PMMA) core/shell nanofibers with a slight change in the setup. To ensure the flow of the melt CBT and avoid clotting in the inner nozzle, a heating tape was wrapped around the entire system. 

### 2.5. Needleless Electrospinning

**Figure 8:**
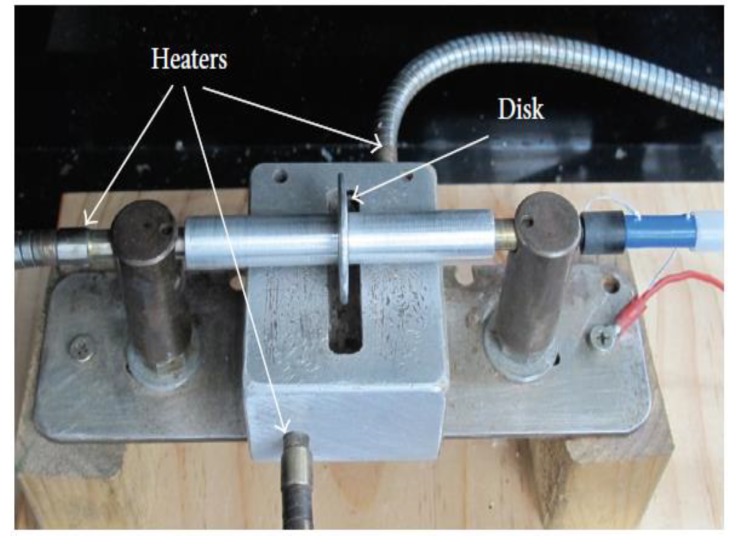
Needleless/disc melt electrospinning, where, the melt polymer is drawn out for the reservoir through the edge of the disc once it rotates (retrieved under the terms and conditions of the Creative Commons) [77].

The needleless melt electrospinning design was first introduced by Fang et al. [77] providing an efficient, continuous and high mass production of fibers as compared with the conventional needle-based electrospinning design. The proposed design as shown in Figure 8, consisted of a melt reservoir, where a rotating disc was partially immersed, drawing out the melt polymer through the edge of the disc. Both the disc and the reservoir were heated separately using cartridge heaters while being connected to the control system to maintain the melt temperature between 280 and 360 °C. The collector was placed above the disc while applying a high voltage and the entire system was inside a plastic box filled with argon. During the experiment, iron and aluminum discs were used as the fiber generators at 16 cm and 75 kV collecting distance and applied voltage, respectively. Fine fibers without beads were produced with 3.31 µm average diameter in the case of using the aluminum disc. On the other hand, the iron disc produced rough surface fibers with some beads at 8.69 µm average diameter. The result signified that the disc material has a major effect on the diameter and the morphology of the fiber diameter, which is quite reasonable since the aluminum has a higher thermal conductivity than iron at the same temperature. It is noteworthy to point out that the electric field intensity had a small effect on thinning the fiber diameter, unlike other studies [29,78,79], which is probably due to the absence of the needle restriction on the Taylor cone volume. Fang’s setup solely depended on the electric field intensity to draw the melt polymer from the surface of the disc since no pump was used, therefore, upon increasing the voltage above 80 kV, more of the melt polymer was drawn away from the disc, in other words, increasing the fiber formation.

An umbellate nozzle-based electrospinning design was introduced by Haoyi Li et al. [80] addressing the factors that affect the distance between the peak of the two adjacent Taylor cones, referred to as the interject distance [81]. The equipment used consisted of a melt inlet, a distributor to ensure a uniform flow on the circumference of the nozzle, an internal and outer surface umbellate nozzle with 16 and 26 mm diameters with 60° cone angle, high power supply, and a circular copper collector with 150 and 3 mm diameter and thickness, respectively. The experimental result showed that the melt viscosity and the applied voltage had the greatest influence on the interject distance. The shortest interject distance at 250 °C achieved on the outer surface nozzle was 1.1 mm at 40 kV, and 10 cm collector distance and 1.4 mm on the internal surface nozzle at 63 kV and 11 cm, while four times the interject distance was reported by Komerek et al. [82]. 

A recent bubble melt electrospinning process was proposed by Li to increase the yield production of microfiber [83]. The melt electrospinning setup was similar to that of Fang except for the rotating disc, where an air pump was used instead. After the polymer melts in the metal container, the pump was turned on and the flow gradually increased until bubbles formed on the surface of the melt polymer. The bubbles burst due to its exposure to high voltage forming 5–45 µm fiber diameter of polyurethane, the reason behind this variation in the fiber diameter is based on each bubble size, viscosity, and its collector distance. Even though, it was challenging to control the production of uniform fibers, the design is considered applicable for mass production.

### 2.6. Other Designs

Several designs were introduced over the past years to enhance the melt electrospinning process and to decrease the fiber diameter, such as Rangkupan and Reneker [84] vacuum electrospinning. Their attempt to spin polypropylene in a vacuum chamber to simulate a space environment enabled them to exert a higher electric field since the vacuum has a higher electrical breakdown than air. The fiber diameter tended to be affected greatly by the applied high voltage and the radiated heat form melt jet such that at 200 kV/m and 300 °C, the spun fibers were in the range of 300 nm to 30 µm. Dalton et al. [85] used circulating hot water to spin low melting point copolymers of poly(ethylene glycol) and (polycaprolactone) (PEG-b-PCL) with different molecular weights. Whereas, Morikawa et al. [86] introduced the wire melt electrospinning process where the geometry of the polymer spin source used was a wire instead of the conventional needle. Despite the simplicity of the method, the usage of the wire resulted in confining the Taylor cone size, which gave rise to decreasing the melt jet. 

Deng et al. [79] introduced a simple and cheap melt electrospinning design to focus the spotlight on the pros for melt electrospinning using a low-melt flow index polymer. This design was composed of 45 steel cylinder and piston, whereas, the capillary was made from stainless steel. A flat wire mesh collector was connected to a high-voltage supply device with a maximum voltage +60 kV and a current of 2 mA. The electrical heating ring and thermal sensor were deployed to melt and measure the temperature of low-density polyethylene (LDPE). To decrease the viscosity of the low-melt flow index LDPE, the spinning temperature was set at the range (315–355 °C) right below the decomposition temperature (360–400 °C). By eliminating the use of the pump, melt polymer was drawn out by the electrostatic force, while the cylinder was fixed in a tilted position. In addition, the electrodes were reversed to prevent interference between the high voltage and temperature sensor. Owing to the mentioned modifications, this design is considered simpler as compared with other reported designs [29,34,53,57,87,88]. The effects on the properties of LDPE were investigated after varying different process parameters. For instance, the higher the temperature, the thinner the fibers formed and since the operating spinning temperature was very high, the fabricated LDPE fibers (<15 µm) were smaller than the fibers produced by Larrondo and Manley [28] with high-density polyethylene (HDPE). Additionally, the electrospun melt fibers showed crystallinity unlike other reported studies [87,88,89]. 

Additionally, Xie et al. [90] investigated the effect of pulsed electric field on the poly(lactic acid) melt, which was first introduced by Baba et al. [91]. Xie reported that the pulsed electric field tended to reduce the fiber diameter as a fine fiber were obtained at 29.8% duty cycle and the method showed progress in the development of fiber structures. Finally, it is worth mentioning the latest emerging technique, melt writing electrospinning (MWE). In terms of process, the MWE technique is a combination of melt electrospinning and 3D printer producing micron and submicron fibers while controlling their deposition in three dimensions [92,93,94]. The key parameters of the MWE are pressure, temperature, collector distance, voltage, and collector speed [95]. A vast range of applications [96,97,98,99,100,101] demonstrate the MWE potential to fabricate scaffolds making it ideal for tissue engineering scaffold development [102].

Table 1 summarizes the different methods of melt electrospinning used to synthesize a wide variety of fibers. In addition, the table demonstrates the difference in process parameters for each example.

## 3. Challenges and Obstacles of Melt Electrospinning 

Although melt electrospinning was first reported in the 1936 by Charles Norton [25], however, it has not been investigated as deeply as solution electrospinning. To illustrate, until 2011, only 0.3% of the publications from electrospinning literatures are found to be focusing on melt electrospinning scaffold design [94]. This is due to notable limitations and challenges in using melt electrospinning such as the relative complexity of the design and the operation process, larger fiber diameter, and few portable commercialized melt electrospinning apparatus. Efforts have been devoted by researchers to tackle the problem of producing fibers with relatively large diameters (tens of microns) [103,104]. However, the setup required for maintaining high temperature, which is added to obtain the desired reduced fiber diameter, is considered as a limitation for the low conductivity and thermo-sensitive natural polymers such as chitosan [79,105,106]. 

In addition, unstable surface tension and spin line cohesive fractures have been reported as challenges in obtaining electrospun fibers of consistent diameter under the submicrometer scale [28,34,107]. Although several attempts have been implemented to improve the productivity of melt electrospun fibers, few commercialized large-scale industrialized apparatus are available for melt electrospinning production nowadays [67,77,108,109]. In spite of the fact, that the small hand-operated Wimshurst generator melt electrospinning setup was designed for in-situ wound dressing, researchers are still struggling to develop a portable melt electrospinning apparatus [110]. Furthermore, one of the important concerns about melt electrospinning is to achieve sufficient flows through small diameter as the flow of the melt through small diameter orifices is limited. This is due to the elevated viscosity of fluid passing to the spinneret, which is tenfold higher as compared with solution electrospinning [79]. Finally, it is worth mentioning that most of electrospinning theories have relied on solution electrospinning so far. Hence, deep investigation studies on melt electrospinning are considered a prerequisite to improve our knowledge about this technique.

## 4. Conclusions

Throughout this review, we can conclude that the electrospinning technique is considered a simple technique with a great ability to produce a huge variety of fibers of different size ranges and applications. Various setup designs have been developed throughout the last two decades for the purpose of enhancing the structure and functionalization of the fabricated fibers. New techniques have been recently articulated such as using the melt writing electrospinning to develop tissue engineering scaffolds. Such novel designs open up the door for more progress and innovation in research to gain additional advantages and applications.

## Figures and Tables

**Table 1:** Different approaches to melt electrospinning with their processing parameters.

Design Method		Polymer	Process Parameters		Fiber Diameter	Ref.
Conventional		PE	T1 = 200–220 °C	ND = 2.2 mm, V = 6 kV/cm & Cd = 1–3 cm	-	[28]
		PP	T1 = 220–240 °C		-	
Multitemperature control		PLA	T1 = 200 °C, T2 = 255 °C, T3 = 80 °C & T4 = 25 °C F = 0.001 mL/min, V = 20 kV, Cd = 10 cm & ND = 0.16 mm		800 nm	[29]
		PP	T1 = 230 °C, T2 = 280–290 °C, T3 = 100–140 °C, T4 = 85–95 °C, V = 10–20 kV, F = 0.002–0.008 mL/min & Cd = 5–7 cm		–	[34]
		PP	T1 = 330–390 °C, T2 = 100–150 °C, T3 = 25 °C, V = 35 kV & Cd = 10–18 cm		~20 µm	[35]
		iPP	T1 = 240 °C, T2 = 180 °C, T3 = room temp, Cd = 2 inch, Cv = 28 kV, Nv = −5 kv & F = 0.001 mL/min		2.4 µm	[36]
		N6	T1 = 270 °C, T2 = 280 °C, T3 = 210–220 °C, F = 0.03 mL/h, Cd = 90 mm, Cv = 29 kV & Nd = 0.26 mm		0.9 µm	[37]
Gas assist		PLA	TM&A = 483 K, Av = 300 m/s, F = 1.67 × 10^–10^ m^3^/s & Cd = 0.09 m		0.18 µm	[42]
		PP	T2 = 260 °C, Av = 30 m/s, nozzle to electrode = 10 cm, V = 35 kV, Cd = 200 nm & RDs = 0–500 rpm		400 nm	[48]
		PLA + 6 wt% ATBC	FA = 25 m/s, V = 40 kV, T = 240 °C & Cd = 9 cm		236 nm	[47]
Laser	spot	PLA	V = 26–30 kV, PL = 13–17 W, Cd = 20 mm & λ = 10.6 µm		712–804 nm	[53]
		EVAL	F = 2–4 mm/s, V = 18–20 kV, Cd = 25 mm, PL = 8–22 W & λ = 10.6 µm		740 nm–2.842 µm	[56]
		PLLA coated with EVOH	F = 10 mm/min, V = 25 kV, PL = 12 w, CD = 5 cm, λ = 10.6 µm & T3 = 40 °C		845 ± 500 nm	[58]
	line	EVOH/Nylon 6/12 sheets	V = 40 kV, PL = 45 W, F = 0.25 mm/min & Cd = 10 mm		800 nm	[67]
		PP/EVOH/PP	Cd = 100 mm, V = 20–70 kV, λ = 10.6 µm & F = 4 mm/min		0.64–1.08 µm	[69]
Coaxial		PEG/PVDF	V = 12 kV, Cd = 17 cm, N1ID = 0.35, N1OD = 0.65 mm, N2ID = 1.05, N2OD = 1.2 mm, FN2 = 1.5 mL/h & FN1 = 0.09–0.24 mL/h		637–911 nm	[71]
Needleless		PP	T = 320 °C, V = 75 kV, Cd = 16 cm		3.31 µm	[77]
		Pp	T = 260 °C, V = 39–63 kV, Cd = 11 cm & ND = 16 mm		14.6–5.3 µm	[80]
		TPU	T = 240 °C	V = 18–25 kV	20 µm	[83]
		PLA	T = 200–250 °C		30 µm	
Others		Pp	T = 300 °C, V = 200 kV/m & vacuum pressure		300 nm–30 µm	[84]
		PEG-b-PCL	V = 20 kV, F = 0.02–5 mL/h & T = 80–90 °C		560 ± 90 nm–16 ± 10.7 µm	[85]
		PCL	T = 100, Cd = 5 cm, Csp = 270 rpm & V = 15–17 kV		1 ± 0.9 µm	[86]
		PCL	Csp = 310–400 mm/min, Cd = 7–13 mm, P = 0.6–1 bar, V = 5.5–7 kV		48.31–75.12 µm	[95]

ND: nozzle diameter, N1ID/ N1OD: inner and outer diameter for inner nozzle, N2ID/ N2OD: inner and outer diameter for outer nozzle, T = temperature, T1: syringe temperature, T2: nozzle temperature, T3: spin area temperature, T4: collector temperature, V = voltage, F/FM: melt flow, FA: air flow, FN1: flow in inner nozzle, FN2: flow in outer nozzle, Cd: collector distance, Csp: collector speed, RDs: rotating disc speed, Cv: collector voltage, Nv: nozzle voltage, TM & A: melt and air temperature, Av: air velocity, PL: laser power, λ: laser wavelength & P: pressure.
